# The Decline in Diffuse Support for National Politics: The Long View on Political Discontent in Britain

**DOI:** 10.1093/poq/nfx020

**Published:** 2017-05-31

**Authors:** Will Jennings, Nick Clarke, Jonathan Moss, Gerry Stoker

**Affiliations:** 1 Will Jennings is a professor of political science and public policy at the University of Southampton, Southampton, UK. Nick Clarke is an associate professor of human geography at the University of Southampton, Southampton, UK. Jonathan Moss is a senior research assistant at the University of Southampton, Southampton, UK. Gerry Stoker is chair of governance at the University of Southampton, Southampton, UK, and Centenary Research Professor of Governance at the Institute for Governance and Policy Analysis, University of Canberra, Canberra, Australia. The authors thank Roger Mortimore and Laurence Stellings and participants at a workshop at New Place, Southampton, for their comments on an earlier version of this paper. The authors also thank YouGov for conducting the October 2014 online survey that is used in their analysis; and they thank the editors and three anonymous reviewers for their useful comments on the manuscript. This work was supported by the UK Economic and Social Research Council for the research project “Popular Understandings of Politics in Britain, 1937–2014” [ES/L007185/1 to N.C., G.S., and W.J.].

## Abstract

This research note considers how to track long-term trajectories of political discontent in Britain. Many accounts are confined to using either survey data drawn from recent decades or imperfect behavioral measures such as voting or party membership as indicators of political disengagement. We instead develop an approach that provides the long view on political disaffection. We first consider time-series data available from repeated survey measures. We next replicate historic survey questions to observe change in public opinion relative to earlier points in time. Finally, we use Stimson’s (1991) dyad-ratios algorithm to construct an over-time index of political discontent that combines data from multiple poll series. This reveals rising levels of political discontent for both specific and diffuse measures of mass opinion. Our method and findings offer insights into the rising tide of disillusionment afflicting many contemporary democracies.

Many claim that contemporary politics is afflicted by a rising tide of political disaffection reflected in negative attitudes toward mainstream politicians and institutions and the rise of electoral support for unconventional or populist candidates and parties (Nye, Zelikow, and King 1997; Pharr and Putnam 2000; Torcal and Montero 2006). The underlying concern is that discontent may make it more difficult for governments to act effectively (Hetherington 2006; Hetherington and Rudolph 2015). Others argue, in contrast, that a degree of distrust of government and politics is healthy in a democracy (Rosanvallon 2008), or that political discontent is not on the rise and that public opinion is characterized by trendless fluctuation rather than clear decline (Norris 2011; Merkel 2014).

A conceptual starting point is David Easton’s (1965) well-known distinction between specific and diffuse support for political systems. The former concerns support for the government of the day, its leaders, and its policies. The latter refers to support for the basic political arrangements of politics and democracy. Trends in diffuse support are the primary focus of our attention. Are we witnessing a level of disillusionment with mainstream politics that is unremarkable and within the bounds of the “normal,” or are we seeing evidence of sustained decline in diffuse support?

Taking a long view of public opinion, however, is not straightforward. Evidence used on either side of the debate to assess trends in the disaffection of citizens with politics is complicated by the limited time frame of the data available. Pharr and Putnam (2000) rely upon a small number of time points or data over just a couple of decades (waves of the World Values Survey during the period between 1981 and 1996). Norris (2011) looks at trends in satisfaction with democracy back to the 1970s (based on data from the Eurobarometer survey from 1973 to 2009).

The puzzle of whether political disaffection has been on the rise is, therefore, confounded by the spottiness and sparseness of available data, in particular prior to the 1970s. Behavioral measures of political engagement, such as voting or party membership, have obvious limits as substitute indicators of political discontent (for example, does non-voting reflect discontent or satisfaction?). Even beyond this, the sorts of expressions of political discontent that have been the subject of survey research in different periods tend to reflect the prevailing concerns and circumstances of the time.

Tracking long-term trajectories of political disaffection in a way that overcomes such data limitations thus poses a substantial methodological challenge. This research note shows that we can address this challenge through use of trend data from repeated survey measures and contemporary replication of historic survey questions, combined with Stimson’s (1991) dyad-ratios algorithm, to draw out trends from a range of related survey questions. Our analysis draws on survey measures of British public attitudes as far back as 1944. Aside from the United States, Britain has more historical survey data pertaining to trust and disaffection than any other country and offers a prominent case for the rise of political discontent (Hay 2007; Stoker 2017).

## Meeting the Challenge: First Steps

**Figure 1.**
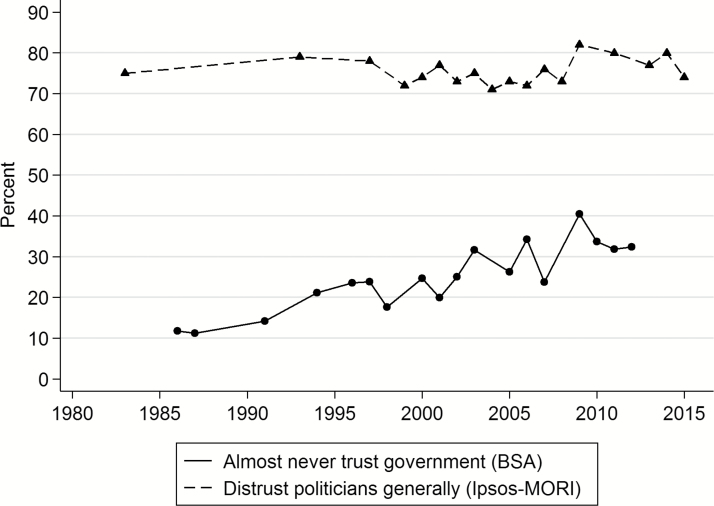
Distrust in British Government and Politicians, 1983–2015.

A more long-term view on diffuse support in British politics can be derived from *trends* in survey questions that have been asked on regular occasions over an extended period. The most widely used measure of diffuse support is a question that has been asked in the British Social Attitudes (BSA) survey since 1986: “How much do you trust British governments of any party to place the needs of the nation above the interests of their own political party?” The percentage of respondents replying “almost never” (rather than “most of the time” or “almost always”) is plotted in figure 1. This reveals a steady decline in trust in government to set partisan interests aside in the wider public interest. Over roughly the same period, Ipsos MORI asked for a range of groups of people, including politicians generally, whether respondents “generally trust them to tell the truth or not.” The percentage of respondents saying they do not trust politicians to tell the truth is also plotted in figure 1. Using this measure, the level of distrust is much higher, but the upward trend is much less pronounced.

This mixed picture shows how the chosen survey measure can shape understanding of long-term trends in the withdrawal of diffuse support. The time frame is also important. If 1983 or 1993 is taken as the starting point in the Ipsos MORI poll series, current levels of distrust appear to have returned to their equilibrium—after a slightly more optimistic period. If 1999 is instead taken as the benchmark, it appears as if there has been a steady erosion of trust in politicians.

**Figure 2.**
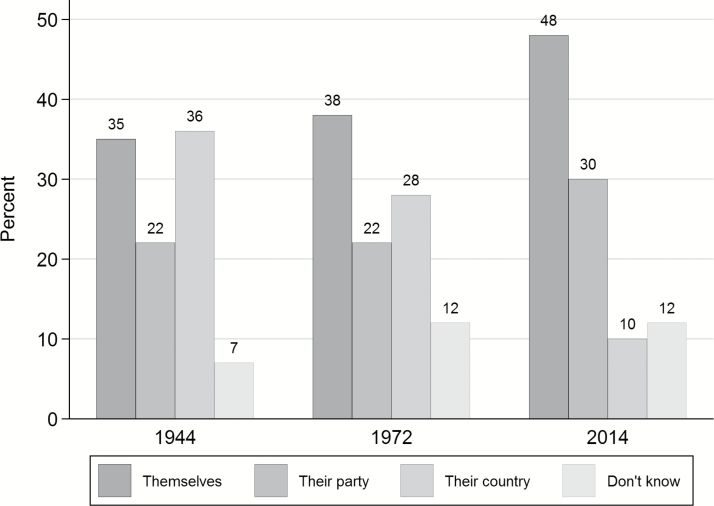
British Politicians Out for Themselves, Their Party, or Their Country, 1944–2014.

An alternative to using trend data is to detect long-term shifts in public attitudes through contemporary replication of historic survey measures. By fielding questions asked at earlier points in time, it is possible to observe change (or stability) in public opinion.^1^ For example, in October 2014 we commissioned an online survey of 2,103 respondents by YouGov, which asked a question originally fielded by Gallup in July 1944 and again in August 1972: “Do you think that British politicians are out merely for themselves, for their party, or to do their best for their country?” The proportion of responses for each is plotted in figure 2.

From observing public opinion on this question over an extended period, certain patterns become apparent. First, there is only a slight increase in the percentage of respondents viewing British politicians as self-seeking between 1944 and 1972 (increasing from 35 to 38 percent), but rather more of a fall in the number believing politicians are out to do the best for their country (from 36 to 28 percent). There is a much bigger shift, however, between 1972 and 2014 in the proportion seeing politicians as out for themselves (rising from 38 to 48 percent) and out for their country (slumping from 28 to 10 percent). What this approach cannot do is fill in the missing gaps for the periods where a survey question has not been fielded.

## Meeting the Challenge: A Longitudinal Measure of Political Discontent

Notwithstanding discontinuities in available data, every repeated survey measure provides *some* information about long-term trends in political disaffection. Observations at different time points indicate the direction of travel of public opinion on that particular measure for a defined period. Trends across multiple measures may exhibit common variance, which is informative about the prevailing mood of public opinion toward politics, politicians, and the system of government.

Using Stimson’s (1991) dyad-ratios algorithm, we construct an index of political discontent based on 37 survey questions, asked 295 times over the period between 1944 and 2016 (in practice, most are from mid-1960s onward, so estimates are not reliable prior to this). This method has been used previously to generate measures of trust in US government (Chanley, Rudolph, and Rahn 2000; Keele 2007). Here, it is used to measure the latent dimension of public expressions of discontent toward politics, politicians, and the political system. Simply, it captures the relative degree to which the public is more or less disaffected with politics. Stimson’s method offers a solution to the problem of irregular and infrequent availability of poll data. The principle behind the dyad-ratios algorithm is intuitive; it uses the ratio of aggregate-level survey responses (“marginals”) to the same question, at different points in time, to derive information about the relative state of public opinion—telling us whether, on average, public attitudes have become more negative or positive toward politics and politicians (see Stimson [1991, appendix 1] and Bartle, Dellepiane-Avallaneda, and Stimson [2011] for discussion of the method).^2^ This extracts the underlying tendency of all survey items relating to political distrust, disaffection, alienation, and so on, analogous to a principal components approach. We use data from a range of sources, including the British Social Attitudes survey, the British Election Study, European Social Survey, Eurobarometer, Hansard Society Audit of Political Engagement, and poll data from Gallup, YouGov, and Ipsos MORI.

Table 1 reports the factor loading of each survey item and the proportion of variance explained by the underlying factor. This reveals that a substantial proportion of variance loads onto a single underlying dimension, indicating the central tendency in public opinion. This accounts for 50 percent of all variance in survey questions on disaffection with politics.^3^ The loading (i.e., correlation with the underlying construct) of a number of survey items is considerable. For instance, the loading of the BSA (no) trust in government series is 0.864, while that for the Ipsos MORI (do not) trust politicians to tell the truth series is somewhat lower, at 0.609, but still substantial. The loading of the Gallup survey question about whether politicians are out for themselves, discussed above, is 0.991. What is striking is that this co-variation of expressions of political disaffection extends across a wide range of measures, some relating to truth-telling, being out of touch with voters, being self-interested or vote-seekers, improper use of public office, or lacking in integrity. Most of these load to a greater or lesser degree onto the underlying construct of political discontent. The prevailing sentiment, or “mood” (Stimson 1991), in public opinion underlies a range of survey responses. This is consistent with commonality observed in expressions of discontent at the individual level (Jennings, Stoker, and Twyman 2016).

**Table 1.** Survey Items and the Measure of Political Discontent

	Political discontent				
Survey item (source)	*N*	Start	End	Factor loading	Standard deviation
Do not trust British governments to place needs of the nation above interests of their own party (BSA)	20	1986	2013	0.864	7.981
Tend not to trust parliament (Eurobarometer)	17	1999	2015	0.935	7.114
Tent not to trust national government (Eurobarometer)	16	1999	2015	0.858	7.005
Do not trust politicians to tell the truth (Ipsos MORI)	18	1983	2015	0.609	3.112
System of governing Britain could be improved (Ipsos MORI, BES, Hansard Society)	19	1973	2015	0.571	7.636
Parties are only interested in people’s votes, not their opinions (BSA)	14	1986	2011	0.652	3.775
Do not trust government ministers to tell the truth (Ipsos MORI)	17	1983	2015	0.364	3.465
Low rating of standards of conduct of public office holders (Committee for Standards in Public Life)	6	2004	2014	0.914	8.770
Those we elect as MPs lose touch with people pretty quickly (BSA)	14	1986	2011	0.341	2.245
Most MPs make a lot of money by using public office improperly (Gallup, YouGov, Ipsos MORI)	5	1985	2009	0.913	8.114
Do not trust politicians of any party to tell the truth in a tight corner (BSA)	15	1994	2013	0.302	1.535
It doesn’t really matter which party is in power (BSA)	7	2001	2011	0.621	3.016
Dissatisfied with way parliament works (Hansard Society)	6	2003	2015	0.665	1.886
People like me have no say in what the government does (BES)	4	1987	2001	0.990	5.054
Most MPs will tell lies if they feel the truth will hurt them politically (Gallup, YouGov)	4	1985	2004	0.957	3.832
MPs use power for personal gain (Ipsos MORI)	4	2004	2013	0.948	9.618
Do not trust MPs to tell the truth (Ipsos MORI)	4	2004	2013	0.776	5.853
Dissatisfied with way MPs are doing job (Hansard Society)	5	2003	2015	0.610	3.098
Distrust in MPs in general (BES)	3	2014	2016	0.999	3.120
British politicians out merely for themselves (Gallup, YouGov)	3	1944	2014	0.991	5.558
Do not trust parliament (European Social Survey)	7	2002	2014	0.409	2.431
Disagree that most MPs have a high personal moral code (Gallup, YouGov, Ipsos MORI)	4	1985	2009	0.715	11.432
MPs put own interests first (Ipsos MORI)	6	1994	2013	0.420	5.121
People like me do not have enough say in way government runs the country (Gallup)	2	1968	1973	1.000	1.500
Do not trust government to tell the truth (Ipsos MORI)	2	2007	2008	1.000	1.994
Do not trust government to act in best interests of country (Ipsos MORI)	2	2007	2008	1.000	2.500
MPs care more about special interests (Gallup)	2	1985	1994	1.000	5.000
Doesn’t really matter which party is in power (BES)	2	2015	2016	1.000	5.119
Do not trust government to put needs of nation above party interests (BES)	2	1987	1997	1.000	1.700
Do not trust parliament (BES)	2	2005	2010	1.000	5.500
Most politicians are in politics only for what they can get out of it personally (BSA)	2	2004	2014	1.000	2.500
Politicians only care about people with money (BES)	3	2014	2016	0.604	1.033
No trust in British politicians (BES)	3	2005	2015	0.601	6.532
Do not trust national politicians (European Social Survey)	7	2002	2014	0.098	2.344
Do not trust local MP to tell truth	4	2004	2013	–0.461	4.023
Bribes and abuse of power for personal gain are widespread among politicians (Eurobarometer)	2	2009	2011	–1.000	1.670
Most of the time cannot trust people in government to do what is right (BSA)	3	2004	2014	–0.836	0.713
First dimension					
Proportion of variance explained	50.4				
*N* of time series	37				
*N* of survey items	295				

Note.—Each survey item is coded as the percentage of respondents giving a negative response about politics, politicians, or the political system (i.e., ordinal measures are recoded as a dichotomous measure of disaffection).

**Figure 3.**
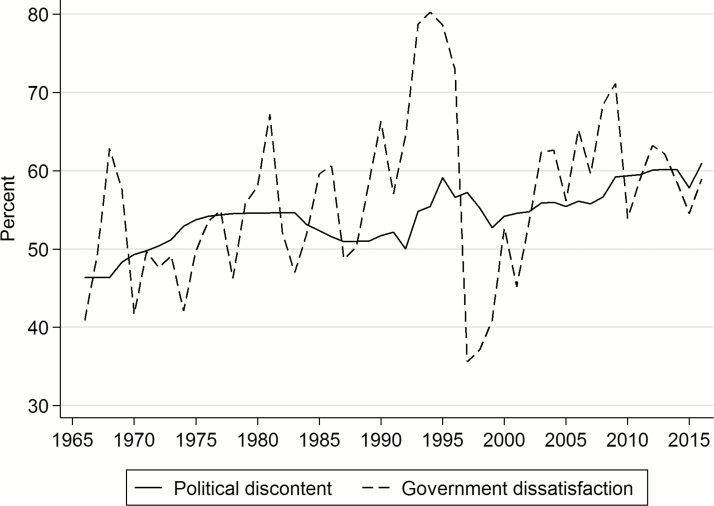
Political Discontent and Government Dissatisfaction in Britain, 1966–2015.

The resulting measure of public discontent with national politics is plotted by the solid line in figure 3. This reveals a steady rise in discontentment from 1966 to 2016.^4^ Combined with the previous evidence on trends and dyadic analysis, we have strong evidence for a sustained growth in discontent with politics over more than half a century. Figure 3 also shows a striking contrast between the slow and steady rise of discontent (that represents a loss of diffuse support) with dissatisfaction with particular governments (specific support). The dashed line in figure 3 plots the annual average of survey measures of government dissatisfaction, from long-running Gallup and Ipsos MORI series (Gallup ceased political polling in Britain in 2001).^5^ While there is a good degree of parallelism in rising levels of dissatisfaction, there are important differences. When it comes to specific support, the trends are more volatile, responding to events and replacement of incumbents by the electorate. Overall, there is still a movement toward a loss of specific support to match the loss of diffuse support. What is driving these changes is a larger question beyond the scope of this research note (see Jennings, Stoker, and Twyman [2016] and Stoker [2017] for some analysis). But it is interesting to note that while polarization has been linked to rising political distrust in the United States (e.g., Nye, Zelikow, and King 1997; Hetherington and Rudolph 2015), if anything the relationship is in the other direction for Britain—where the parties have depolarized since the 1980s.^6^

## Conclusions

Our analysis resolves the question of whether political discontent has increased over time, in the case of Britain. Extensive survey evidence between 1944 and the present has been drawn upon to assess *relative* changes in attitudes about politics, measured at two or more points in time. Specifically, evidence from time-series trends and contemporary replication of historic survey questions has been inspected and the dyad-ratios algorithm used to construct a longitudinal measure of political discontent that captures the underlying dimension of popular expressions of disaffection with politics. Together, these findings support the claim of rising discontentment among citizens, and of the withdrawal of diffuse support. We can be clear that there was no golden age of democracy, as a degree of public skepticism about the political system appears to have been present throughout. We are also able to come down against the “trendless fluctuation” thesis (see Norris [2011]) for Britain, at least if the full postwar period is considered.

Our analysis provides researchers in other countries with some possible clues about how debates over the long-term trajectory of anti-politics might be resolved in the face of data scarcity and limited time frames of analysis. Establishing the temporal scope of anti-politics is also a prerequisite for explaining it (Nye, Zelikow, and King 1997). We offer a step forward. Given evidence of long-term decline in diffuse support, explanations that reflect long-term trends in politics, society, and media appear to fit best. Taking the long view thus offers a promising way of understanding the populist challenge that is facing many contemporary democracies.

## Supplementary Data

Supplementary data are freely available at *Public Opinion Quarterly* online. Data and supporting materials necessary to reproduce the empirical results are available at http://dx.doi.org/10.7910/DVN/Q3OM0J

## Supplementary Material

File 005

## Notes

1 One possible constraint on this strategy is the degree to which questions are context or time specific. A further limitation is the extent to which point estimates derived from surveys using different methodologies are directly comparable, though these can provide important information on the direction and approximate degree of change.

2 Each survey item can be expressed as the ratio of feelings toward politics at two points in time: a “dyad.” This ratio provides an estimate of relative political discontent, for a given question, in years *t+i* and *t+j*:

$$P_{ij}=\frac{X_{t+i}}{X_{t+j}}$$

This enables recursive estimation of the index of political discontent for each survey item for each time period based on all data available. Because there are multiple estimates of political discontent (i.e., there are multiple survey items) and they are not all equivalent indicators of the latent construct, the dyad-ratios algorithm estimates the squared correlation of each series with the underlying dimension and uses this to weight the series (Bartle, Dellepiane-Avellaneda, and Stimson 2011, p. 269). This correlation is interpretable as a factor loading, and is reported below for selected survey items.

3 Sensitivity analysis, dropping one survey item at a time from the measure, indicates that it is highly robust to the available data (i.e. the average correlation with the measure is 0.99 and the proportion of variance explained ranges from 48 to 54 percent). Estimation of the underlying factor is not contingent on inclusion or exclusion of particular data series.

4 The line of best fit for a linear regression of our measure of political discontent as a function of time indicates that it has been growing at a rate of 0.2 points per year over this fifty-year period.

5 The Gallup version of the question asked, “Do you approve or disapprove of the Government’s record to date?” while the Ipsos MORI question is worded, “Are you satisfied or dissatisfied with the way … the Government is running the country?” The correlation of these series for the overlapping period is equal to 0.98 (*p* = 0.000).

6 The correlation between political discontent and elite polarization (as measured by policy distance between party platforms) is not statistically significant for the twelve election years between 1966 and 2015 (*r* = –0.247, *p* = 0.438).

## References

[CIT0001] Bartle John Dellepiane-Avellaneda Sebastian Stimson James A. 2011 “The Moving Centre: Preferences for Government Activity in Britain, 1950–2005.” British Journal of Political Science 41:259–85.

[CIT0002] Chanley Virginia A., Thomas J. Rudolph Rahn Wendy M. 2000 “The Origins and Consequences of Public Trust in Government: A Time Series Analysis.” Public Opinion Quarterly 64:239–56. 11114267 10.1086/317987

[CIT0003] Easton David 1965 *A Systems Analysis of Political Life*. New York: Wiley.

[CIT0004] Hay Colin 2007 *Why We Hate Politics*. Cambridge: Polity Press.

[CIT0005] Hetherington Marc 2006 *Why Trust Matters: Declining Political Trust and the Demise of American Liberalism*. Princeton, NJ: Princeton University Press.

[CIT0006] Hetherington Marc Rudolph Thomas 2015 *Why Washington Won’t Work: Polarization, Political Trust, and the Governing Crisis*.Chicago: University of Chicago Press.

[CIT0007] Jennings Will Stoker Gerry Twyman Joe 2016 “The Dimensions and Impact of Political Discontent in Britain.” Parliamentary Affairs 69:876–900.

[CIT0008] Keele Luke 2007 “Social Capital and the Dynamics of Trust in Government.” American Journal of Political Science 51:241–54.

[CIT0009] Merkel Wolfgang 2014 “Is There a Crisis of Democracy?” Democratic Theory 1:11–25.

[CIT0011] Norris, Pippa. 2011 *Democratic Deficit: Critical Citizens Revisited*.Cambridge: Cambridge University Press.

[CIT0012] Nye Joseph S. Jr. Zelikow Philip D. King David C. 1997 *Why People Don’t Trust Government*.Cambridge, MA: Harvard University Press.

[CIT0013] Pharr Susan J. Putnam Robert 2000 *Disaffected Democracies: What’s Troubling the Trilateral Countries?* Princeton, NJ: Princeton University Press.

[CIT0014] Rosanvallon Pierre 2008 *Counter-Democracy: Politics in an Age of Distrust*. Cambridge: Cambridge University Press.

[CIT0015] Stimson James A 1991 *Public Opinion in America: Moods, Cycles, and Swings*.Boulder, CO: Westview.

[CIT0016] Stoker Gerry 2017 *Why Politics Matters: Making Democracy Work*. 2nd ed London: Palgrave.

[CIT0017] Torcal Mariano Ramón Montero José 2006 *Political Disaffection in Contemporary Democracies: Social Capital, Institutions and Politics*.London: Routledge.

