# Supplementary material for: The Decline in Diffuse Support for National Politics: The Long View on Political Discontent in Britain
Source: Public Opin Q. 2017 May 31;81(3):748–58. doi: 10.1093/poq/nfx020 (PMC5927329; doi:10.1093/poq/nfx020)
Supplement: File 005 [file nfx020_suppl_poq-16-0116-file005.pdf]

Appendix

Table A1. Survey data on trust in politicians/government (Figure 1)

Year	Do not trust politicians generally to tell the truth	Almost never trust British governments
	Ipsos MORI	British Social Attitudes Survey
1983	75	n/a
1986	n/a	12
1987	n/a	11
1991	n/a	14
1993	79	n/a
1994	n/a	21
1996	n/a	24
1997	78	24
1998	n/a	18
1999	72	n/a
2000	74	25
2001	77	20
2002	73	25
2003	75	32
2004	71	n/a
2005	73	26
2006	72	34
2007	76	24
2008	73	n/a
2009	82	40
2010	n/a	34
2011	80	32
2012	n/a	32
2013	77	n/a
2014	80	n/a
2015	74	n/a

Q. "Now I will read out a list of different types of people. For each, would you tell me whether you generally trust them to tell the truth or not? Politicians generally." (Ipsos MORI)

Q. "How much do you trust British governments of any party to place the needs of the nation above the interests of their own political party? Almost never." (British Social Attitudes Survey)

Table A2. Gallup/YouGov survey data (Figure 2)

Year	Pollster	Sample	Fieldwork	Them-selves	Their party	Their country	Don't know
1944	Gallup	n/a	July	35	22	36	7
1972	Gallup	n/a	August	38	22	28	12
2014	YouGov	2,103	October 20-21	48	30	10	12

Q. "Do you think that British politicians are out merely for themselves, for their party, or to do their best for their country?" (Gallup/YouGov)

Table A3. Dyad ratios algorithm output (Stimson: WCalc 6.1)

295 records after date scan

Period: 1944 to 2016, 73 Time Points

Number of Series: 37

Exponential Smoothing: On

Iteration History: Dimension 1

Iter	Convergence	Criterion	Items	Reliability	AlphaF	AlphaB
1	.2321	.001	37	.921	.500	.500
2	.0169	.001	37	.915	.500	.505
3	.0045	.001	37	.912	.503	.505
4	.0011	.001	37	.912	.505	.505
5	.0006	.001	37	.911	.504	.504

Loadings and descriptive variable information

Vn	Variable	Cases	Dim 1 Loading	Dim 2 Loading	Mean	Std Deviation
1	govtrust_bsa	20	.864	.000	24.715	7.981
28	eb_trustprl	17	.935	.000	59.327	7.114
29	eb_trustgov	16	.858	.000	64.928	7.005
15	trust_mori2	18	.609	.000	75.611	3.112
33	h_govsys	19	.571	.000	62.672	7.636
16	bsa_votes	14	.652	.000	72.907	3.775
14	trust_mori1	17	.364	.000	73.412	3.465
37	cspl_pubstd	6	.914	.000	21.500	8.770
17	bsa_MPs	14	.341	.000	74.043	2.245
7	improp_g	5	.913	.000	57.600	8.114
19	bsa_poltrust	15	.302	.000	91.920	1.535
20	bsa_parties	7	.621	.000	68.214	3.016
35	h_parlsat	6	.665	.000	34.333	1.886
22	bes_nosay	4	.990	.000	51.425	5.054
9	pollies_g	4	.957	.000	82.250	3.832
11	mpgain_m	4	.948	.000	57.000	9.618
12	trustmps_m	4	.776	.000	68.501	5.853
34	h_mpssat	5	.610	.000	38.000	3.098
26	bes_mpstrust	3	.999	.000	52.736	3.120
2	pols_g	3	.991	.000	40.333	5.558
32	ess_trustparl	7	.409	.000	48.579	2.431
8	polmor_g	4	.715	.000	54.250	11.432
4	pols_mori	6	.420	.000	53.667	5.121
3	efficacy_g	2	1.000	.000	69.500	1.500
5	govtrust_m	2	1.000	.000	69.006	1.994
6	govtrust2_m	2	1.000	.000	45.500	2.500
10	spint_g	2	1.000	.000	72.000	5.000
21	bes_parties	2	1.000	.000	23.182	5.119
23	bes_govtrust	2	1.000	.000	10.900	1.700
25	bes_wmtrust	2	1.000	.000	35.500	5.500
36	bsa_pols	2	1.000	.000	43.500	2.500
27	bes_polmoney	3	.604	.000	58.553	1.033
24	bes_polstrust	3	.601	.000	52.000	6.532
31	ess_trustpol	7	.098	.000	61.850	2.344
13	trustown_m	4	-.461	.000	40.750	4.023
30	eb_polcor	2	-1.000	.000	59.710	1.670
18	bsa_govtrust2	3	-.836	.000	61.667	.713

Dimension 1 Information
Eigen Estimate 1.77 of possible 3.51
Pct Variance Explained: 50.39

Weighted Average Metric: Mean: 51.89 St. Dev: 4.61

Table A4. Data series (Figure 3)

Year	Political discontent	Government dissatisfaction
1966	46.371	40.9091
1967	46.371	49.2727
1968	46.371	62.8333
1969	48.324	57.6667
1970	49.293	41.6667
1971	49.773	49.6667
1972	50.408	47.6667
1973	51.183	49.1667
1974	52.915	42.0909
1975	53.774	49.7500
1976	54.200	53.5833
1977	54.411	54.9286
1978	54.516	46.2632
1979	54.568	55.8050
1980	54.594	58.0455
1981	54.607	67.2174
1982	54.613	52.1667
1983	54.617	47.0476
1984	53.108	52.0455
1985	52.360	59.6250
1986	51.591	60.6250
1987	50.959	48.7391
1988	51.004	50.2500
1989	51.026	58.2500
1990	51.708	66.3292
1991	52.160	57.0458
1992	50.030	64.5391
1993	54.840	78.7000
1994	55.437	80.2696
1995	59.140	78.6542
1996	56.609	72.9708
1997	57.263	35.6130
1998	55.277	37.1042
1999	52.740	40.8083
2000	54.187	52.7000
2001	54.560	45.2000
2002	54.775	53.3636
2003	55.903	62.4167
2004	56.005	62.6667
2005	55.468	56.2000
2006	56.109	65.2727
2007	55.782	59.6667
2008	56.669	68.5000
2009	59.220	71.1667
2010	59.358	53.9000
2011	59.533	58.9167
2012	60.090	63.2500
2013	60.179	62.1667
2014	60.123	58.5000
2015	57.823	54.5455
2016	60.958	59.0000
